# Advancing biological super-resolution microscopy through deep learning: a brief review

**DOI:** 10.52601/bpr.2021.210019

**Published:** 2021-08-31

**Authors:** Tianjie Yang, Yaoru Luo, Wei Ji, Ge Yang

**Affiliations:** 1 Institute of Biophysics, Chinese Academy of Sciences, Beijing 100101, China; 2 College of Life Sciences, University of Chinese Academy of Sciences, Beijing 100049, China; 3 Laboratory of Computational Biology and Machine Intelligence, School of Artificial Intelligence, University of Chinese Academy of Sciences, Beijing 100190, China; 4 National Laboratory of Pattern Recognition, Institute of Automation, Chinese Academy of Sciences, Beijing 100190, China

**Keywords:** Super-resolution microscopy, Image super-resolution, Deep learning, Image reconstruction, Fluorescence microscopy

## Abstract

Biological super-resolution microscopy is a new generation of imaging techniques that overcome the ~200 nm diffraction limit of conventional light microscopy in spatial resolution. By providing novel spatial or spatiotemporal information on biological processes at nanometer resolution with molecular specificity, it plays an increasingly important role in biomedical sciences. However, its technical constraints also require trade-offs to balance its spatial resolution, temporal resolution, and light exposure of samples. Recently, deep learning has achieved breakthrough performance in many image processing and computer vision tasks. It has also shown great promise in pushing the performance envelope of biological super-resolution microscopy. In this brief review, we survey recent advances in using deep learning to enhance the performance of biological super-resolution microscopy, focusing primarily on computational reconstruction of super-resolution images. Related key technical challenges are discussed. Despite the challenges, deep learning is expected to play an important role in the development of biological super-resolution microscopy. We conclude with an outlook into the future of this new research area.

## INTRODUCTION

### Biological super-resolution microscopy

**Figure 1:**
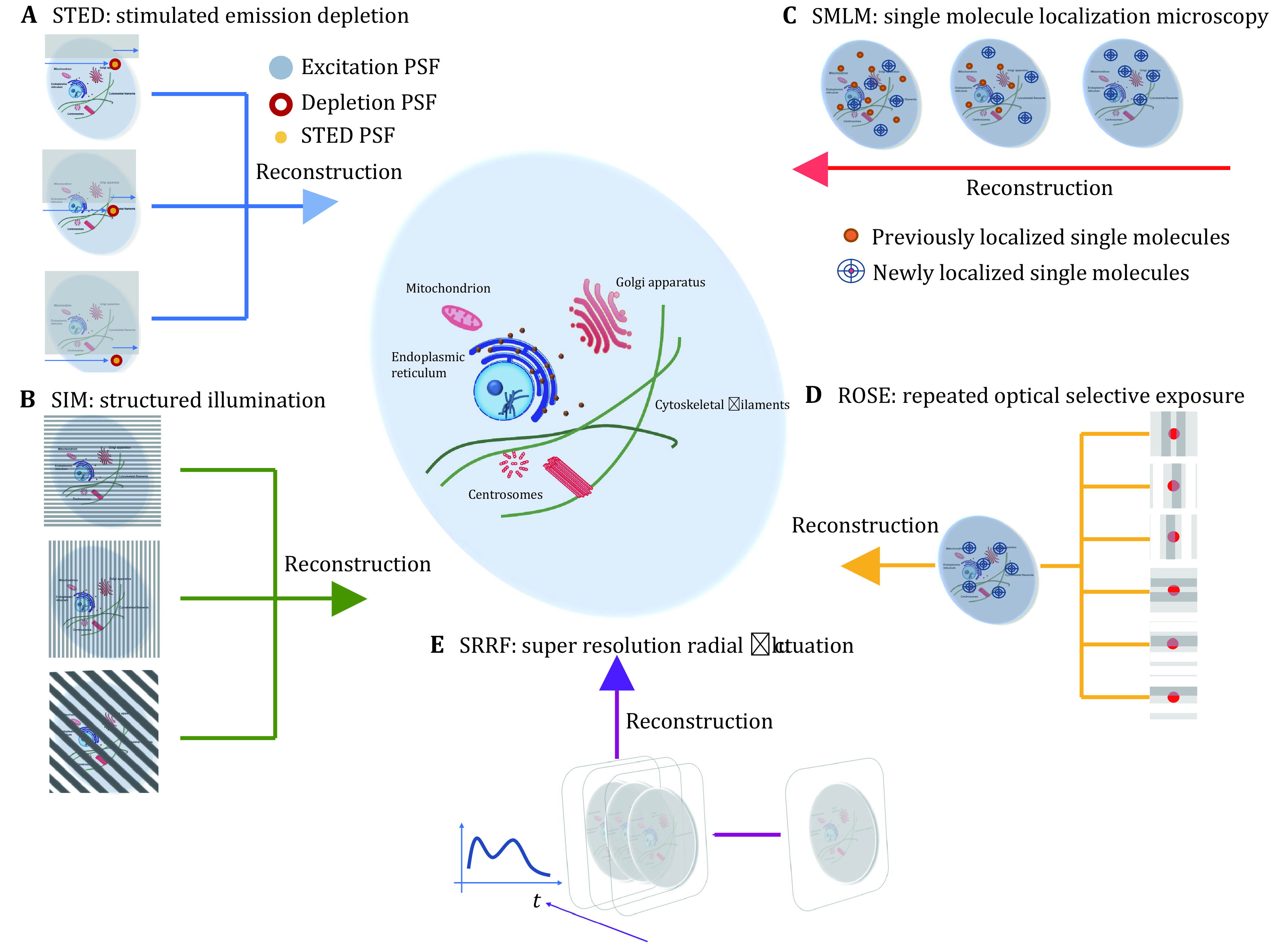
Visualizing nanoscale structures of cells using different super-resolution microscopy techniques. **A** STED: image acquisition is performed by scanning the field-of-view. Final resolution is determined by the size of the STED PSF (point spread function). **B** SIM: a sequence of raw images is acquired under various structured illumination patterns. Raw images are then computationally processed to reconstruct a single super-resolution image. **C** SMLM: single molecules are localized frame by frame and accumulated to form the final super-resolution image. **D** ROSE: single molecules are localized under structured illumination and accumulated to form the final super-resolution image. **E** SRRF: fluctuation of fluorophore signals is computationally analyzed to enhance spatial resolution

Fluorescence microscopy is a light microscopy technique that plays a critical role in biomedical sciences by capturing spatial or spatiotemporal information of biological processes (Lichtman and Conchello 2005). Its molecular specificity, low invasiveness, and multiplex capability make it a powerful tool for studying structure and function of biological processes in space and time at the molecular level under physiological conditions (Giepmans *et al*. 2006). However, the spatial resolution of conventional fluorescence microscopy is limited by the diffraction of visible light to ~200 nm. Under this resolution limit, many important molecular level details of biological processes are indistinguishable. Super-resolution microscopy overcomes this limit (Fig. 1), routinely reaching spatial resolutions in the range of 20–70 nm (Sahl *et al*. 2017; Sigal *et al*. 2018; Valli *et al*. 2021), with some techniques reaching spatial resolutions of <10 nm in certain applications (Balzarotti *et al*. 2017; Gu *et al*. 2019). Depending on their modes of image formation, the wide variety of biological super-resolution microscopy techniques generally fall under two categories: patterned illumination and single-molecule localization.

Super-resolution by patterned illumination was pioneered by stimulated emission depletion (STED) microscopy (Fig. 1A), which uses an intense doughnut-shaped depletion laser beam to create an emission profile smaller than the diffraction limit (Hell and Wichmann 1994; Klar *et al*. 2000). However, strong depletion illumination causes photobleaching and phototoxicity. A similar but more general photoswitching-based technique called reversible saturable optical linear fluorescence transitions (RESOLFT) was developed later, allowing substantially reduced depletion laser intensities (Hofmann *et al*. 2005). Resolutions of these two techniques typically reach tens of nanometers. Under STED and RESOLFT, image acquisition requires point scanning of the field-of-view (FOV) (Fig. 1A). In comparison, structured illumination microscopy (SIM) (Gustafsson 2000) is a widefield-based technique that uses patterned illumination to increase the spatial frequencies that can be captured (Fig. 1B). It can use conventional fluorophores to image a large FOV on a millisecond timescale (Guo *et al*. 2018; Li *et al*. 2015). However, it can only reach ~100 nm in spatial resolution. SIM using nonlinear structured illumination and photoswitchable proteins (NL-SIM) can further improve the resolution to ~50 nm (Rego *et al*. 2012), but to reach a higher resolution remains challenging. Under STED and RESOLFT, super-resolution images are directly acquired by point-scanning and photo-switching at defined spatial coordinates (Sahl *et al*. 2017). Under SIM and NL-SIM, super-resolution images are reconstructed through computational processing of acquired raw images.

Super-resolution by single-molecule localization, often referred to as single-molecule localization microscopy (SMLM), differentiates single switchable fluorophores within the diffraction limit by their blinking events (Fig. 1C). Stochastic optical reconstruction microscopy (STORM) (Rust *et al*. 2006) and photoactivation localization microscopy (PALM) (Betzig *et al*. 2006) are two representatives. STORM uses switchable organic dyes while PALM uses photoactivatable proteins. SMLM can reach a spatial resolution of ~20–30 nm. However, blinking events under SMLM occur at random spatial coordinates. Typically, thousands of raw images or more need to be collected so that enough localized single molecules can be accumulated to faithfully reconstruct the real geometry and fluorescence signal distributions of samples. For this reason, SMLM techniques require long acquisition time, and their low temporal resolution severely limits their applications in live-cell imaging (Sigal *et al*. 2018).

Patterned illumination-based and single-molecule localization-based super-resolution strategies have complementary technical strengths. Recently, several designs have been developed to combine their strengths by integrating the two strategies. In nanoscopy with minimal photon fluxes (MINFLUX), stochastic single-molecule photoswitching is combined with patterned illumination-based localization to reach a resolution of ~1 nm (Balzarotti *et al*. 2017). In repetitive optical selective exposure (ROSE), stochastic single-molecule photoswitching is combined with patterned illumination-based excitation to reach a lateral resolution of ~5 nm (Gu *et al*. 2019) and an axial resolution of ~2 nm (Fig. 1D) (Gu *et al*. 2021). For both techniques, single molecules are computationally localized for the reconstruction of super-resolution images, same as for STORM and PALM.

The super-resolution microscopy techniques introduced so far require specialized optics, specialized fluorophores, or both. Different from these techniques, several computational super-resolution techniques overcome the diffraction limit by analyzing random fluctuations of fluorophore signals. Super-resolution optical fluctuation imaging (SOFI) (Dertinger *et al*. 2009) and super-resolution radial fluctuations (SRRF) (Fig. 1E) (Gustafsson *et al*. 2016) are two representatives. They can be combined with other super-resolution techniques such as STORM and PALM or conventional widefield and confocal microscopy. Reconstruction of super-resolution images using these techniques requires computational processing of acquired raw images. Overall, along with the representative super-resolution microscopy techniques introduced above, many variants have been developed over the past two decades. See *e*.*g*., Sage *et al*. (2019) for a case study on some of these variants. Comprehensive reviews of super-resolution microscopy techniques can be found in *e*.*g*., Sahl *et al*. (2017), Sigal *et al*. (2018) and Valli *et al*. (2021).

Different super-resolution microscopy techniques may differ substantially in their image formation and acquisition. However, from a computational point of view, there are important commonalities in their image reconstruction. In this review, we focus on computational reconstruction of super-resolution microscopy images. For the representative super-resolution microscopy techniques introduced above, Table 1 summarizes and compares the principles and performance goals of their image reconstruction.

**Table 1:** Comparison of image reconstruction for representative super-resolution techniques

Modality		Principle of image reconstruction	Main performance goals for image reconstruction
Image formation	Representative techniques		
Patterned illumination	STED, RESOLFT	Localization of fluorophores at deterministic coordinates; Full frames acquired by point scanning	To maximize spatial and temporal resolution To minimize light exposure
	SIM, NL-SIM	Full frames reconstructed from computational processing of raw widefield images	To maximize spatial and temporal resolution To minimize reconstruction artifacts
Single-molecule localization	STORM, PALM	Localization of single fluorophores through model fitting at stochastic coordinates; Full frames reconstructed through data accumulation	To maximize spatial and temporal resolution To minimize light exposure
Single-molecule fluctuation	SOFI, SRRF	Localization of fluorophores through fluctuation analysis; Full frames reconstructed from computational process of raw image sequences	To maximize spatial and temporal resolution To minimize reconstruction artifacts
Pattern illumination + Single-molecule localization	MINFLUX, ROSE	Localization of single fluorophores at deterministic (MINFLUX) or stochastic (ROSE) coordinates; Full frames reconstructed through scanning (MINFLUX) or data accumulation (ROSE)	To maximize spatial and temporal resolution To minimize light exposure

### Deep learning for image processing and computer vision

Deep learning refers to a class of machine learning or artificial intelligence techniques that compute using artificial neural networks with many layers, often called deep neural networks (DNNs) (LeCun *et al*. 2015). Honored by the 2018 ACM Turing Award, it has revolutionized how we analyze and understand images and has been used with tremendous success in virtually all kinds of image processing and computer vision tasks, such as image classification (Rawat and Wang 2017), object detection (Liu *et al*. 2020; Zhao *et al*. 2019), image segmentation (Garcia-Garcia *et al*. 2017), object tracking (Ciaparrone *et al*. 2020; Li *et al*. 2018), image registration (Fu *et al*. 2020; Haskins *et al*. 2020), image denoising (Tian *et al*. 2020), and image synthesis (Shorten and Khoshgoftaar 2019; Wang *et al*. 2019b). The most commonly used types of DNNs for such tasks are convolutional neural networks (LeCun *et al*. 2015), and for image sequences, recursive neural networks (Yu *et al*. 2019). Other types of neural networks such as graph neural networks (Wu *et al*. 2021; Zhou *et al*. 2020) have also been used for various applications. Most of the deep learning-based image processing and computer vision techniques are developed initially for natural images.

The first step in solving an image processing or computer vision problem using deep learning often is to decide on a learning strategy, such as supervised learning, semi-supervised learning, or unsupervised learning (Karhunen *et al*. 2015; Schmarje *et al*. 2021; van Engelen and Hoos 2020). The decision is often based on the availability of labeled training data and the cost of producing new labeled training data. However, training with unlabeled data can help prevent overfitting (Karhunen *et al*. 2015; Schmarje *et al*. 2021; van Engelen and Hoos 2020). DNNs are trained with fully labeled data in supervised learning, partially labeled data in semi-supervised learning, and unlabeled data in unsupervised learning.

**Figure 2:**
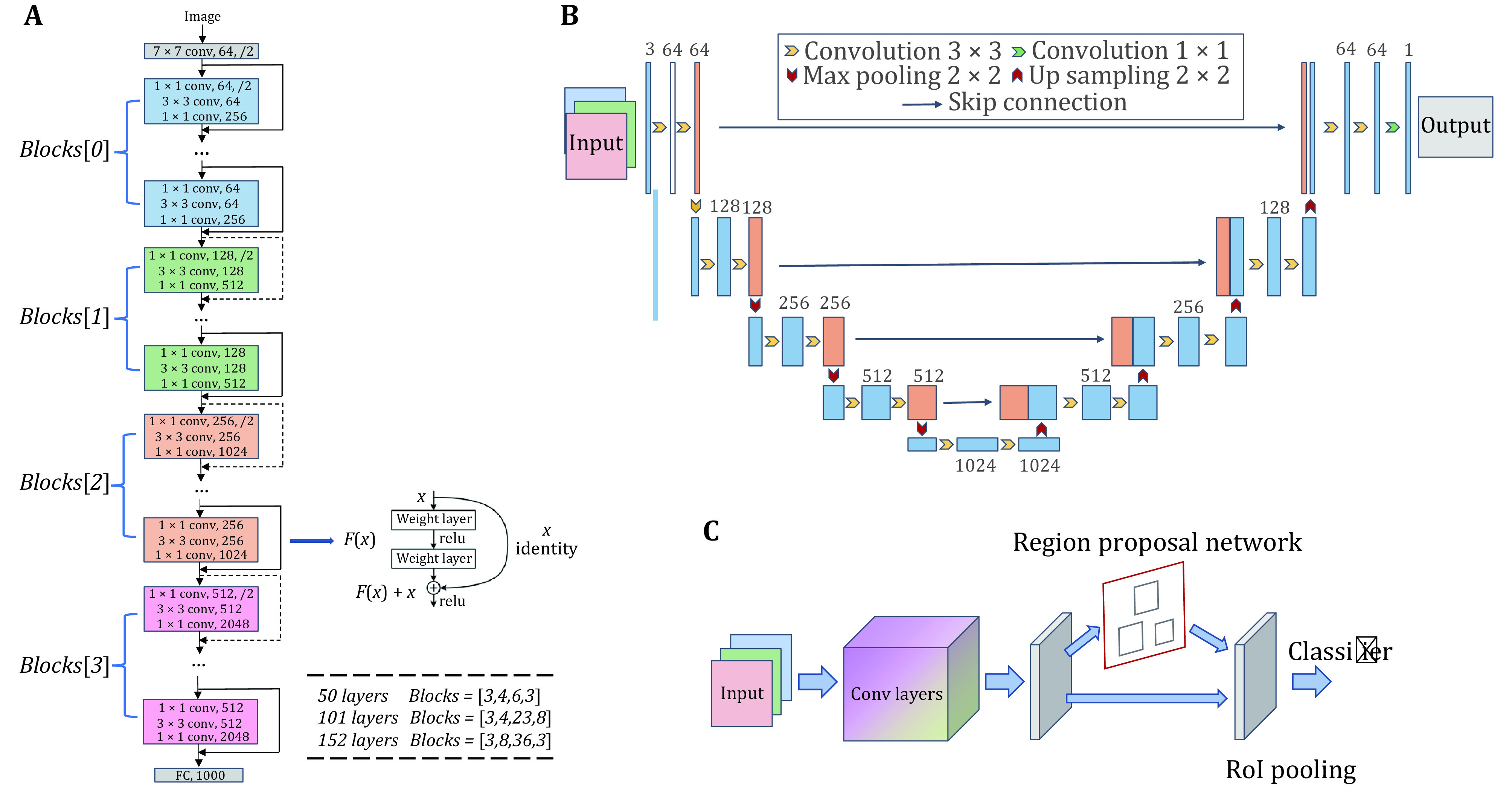
Architectures of representative deep neural networks. **A** ResNet: a feedforward architecture with residual blocks, often used for image classification. **B** U-Net: an encoder-decoder architecture with skip connections at different scales, often used for image segmentation. **C** Faster R-CNN: a two-stage detector architecture with a region proposal network that shares full scale image features with the detection network, often used for object detection

The next step often is to choose an existing DNN architecture or to develop a new or custom DNN architecture, also referred to as a model, for the best performance. Many DNN architectures have been developed over the past decade (Fig. 2). In image classification, for example, the ImageNet Large Scale Visual Recognition Challenge (ILSVRC) (Russakovsky *et al*. 2015) has played a particularly important role in driving the development of new models and in starting the deep learning revolution. Representative models coming out of this competition include AlexNet (Krizhevsky *et al*. 2012), VGG (Simonyan and Zisserman 2014), Inception (GoogLeNet) (Szegedy *et al*. 2015), and ResNet (Fig. 2A) (He *et al*. 2016), to name a few. These networks utilize deep architectures with many layers to improve prediction accuracy and ingenious layer connections to solve the vanishing gradient problem in training deep networks. VGG, for example, utilizes a stack of small (3 × 3) convolution filters to increase network depth without increasing model parameters. GoogLeNet designs an inception module to improve the utilization of computing resources inside very deep and wide architectures. ResNet utilizes residual mapping to address the vanishing gradient problem in deep network training. In object detection, image objects are located and classified by assigning rectangular bounding boxes. One-stage detectors refer to models that combine localization and classification into one step. Representative one-stage detectors include YOLO (Redmon *et al*. 2016), SSD (Liu *et al*. 2016), RetinaNet (Lin *et al*. 2017) and CornerNet (Law and Deng 2018). Two-stage detectors separate object localization and classification into two steps. Representative two-stage detectors include R-CNN (Girshick *et al*. 2014), Fast R-CNN (Girshick 2015), Faster R-CNN (Fig. 2C) (Ren *et al*. 2015), R-FCN (Dai *et al*. 2016) and Mask R-CNN (He *et al*. 2017). Compared to two-stage detectors, one-stage detectors run faster but have lower accuracy. In contrast, two-stage detectors achieve higher accuracy but are typically slower than one-stage detectors. Therefore, it is important to find the right trade-off between speed and accuracy. In semantic image segmentation, individual pixels belonging to the same object are grouped and assigned the same semantic label. Representative models include fully convolutional network (FCN) (Long *et al*. 2015), SegNet (Badrinarayanan *et al*. 2017), and U-Net (Fig. 2B) (Ronneberger *et al*. 2015). Encoder-decoder networks generally perform well in segmentation tasks. For example, U-Net uses a symmetric architecture that concatenates feature maps between its encoder and its decoder. SegNet uses pooling indices to upsample in its decoder for better performance. More recent segmentation models use multiscale image features. Representative models include Pyramid Scene Parsing Network (PSPN) (Zhao *et al*. 2017), Adaptive Pyramid Context Network (APC-Net) (He *et al*. 2019), Multi-Scale Context Intertwining (MSCI) (Lin *et al*. 2018) and High-Resolution Network (HRNet) (Wang *et al*. 2020a). Most recently, the transformer architecture (Khan *et al*. 2021; Wolf *et al*. 2019), originally developed for natural language processing, has found substantial success in image processing and computer vision tasks such as image classification (Kolesnikov *et al*. 2019; Liu *et al*. 2021). It uses a self-attention mechanism to address the problem of long-term dependency. Although it achieves good performance, it also requires substantially more computing resources than conventional convolutional neural networks.

The selection or development of a DNN model is usually accompanied by the selection or development of an application-specific loss function (Jadon 2020; Wang *et al*. 2020b). Training of DNNs is essentially a process that optimizes their connection weights to minimize or maximize the loss function, often referred to as the cost function in optimization. A variety of optimization methods have been developed for network training (Le *et al*. 2011; Sun 2019). Configuration parameters used in the training of DNNs, called hyperparameters, require tuning (Yang and Shami 2020; Yu and Zhu 2020). Overall, in assessing a deep learning technique, key components to be examined include its learning strategy, network architecture, loss function, and training data. Its optimization method and hyperparameters are key to its implementation.

### Deep learning for processing fluorescence microscopy images

In addition to its tremendous success in processing natural images, deep learning has also found great success in processing fluorescence microscopy images (Belthangady and Royer 2019; Gupta *et al*. 2019; Moen *et al*. 2019; Xing *et al*. 2018). From a user’s perspective, deep learning techniques offer at least two important advantages over traditional image processing techniques. First, they offer superior performance. A striking example is the synthesis of realistic fluorescence microscopy images using generative adversarial networks (GANs) (Wang *et al*. 2019b). See *e*.*g*., Feng *et al*. (2019). It is not feasible for traditional image synthesis techniques to achieve the same level of fidelity. Second, deep learning techniques are more user-friendly. Once DNNs are properly trained, they can be used with little or no parameter tuning. In contrast, parameter tuning is usually required for traditional image processing techniques.

Despite the great success of deep learning in processing both natural images and fluorescence microscopy images, it is important to note the differences between these two types of images. First, fluorescence microscopy images have simpler structures and semantics than natural images. For each color (*i*.*e*. wavelength) channel, the semantic label for each pixel is binary, either foreground or background, and the image background is composed of structure-free regions of noise. Second, fluorescence microscopy images have greater pixel depths and wider dynamic ranges than natural images. Third, fluorescence microscopy images have noise properties that differ substantially from those of natural images (Zhang *et al*. 2019). Fourth, blurring of image objects is common in fluorescence microscopy images because of the limited depth-of-field. Overall, it is essential to consider these distinct properties in developing deep learning-based processing techniques for fluorescence microscopy images. It is also essential to consider the technical limitations of deep learning techniques, which are discussed later.

## DEEP LEARNING FOR RECONSTRUCTION OF SUPER-RESOLUTION MICROSCOPY IMAGES

Super-resolution microscopy techniques are defined by their capability to overcome the diffraction limit in spatial resolution. However, spatial resolution is not the only performance metric required by real-world applications. Other important performance metrics may include, *e*.*g*., temporal resolution, duration of image acquisition, and area of image acquisition (*i*.*e*., FOV). In addition to different performance requirements, super-resolution microscopy techniques are subject to technical constraints that define their performance envelopes. For example, SMLM techniques such as STORM and PALM are limited in their temporal resolutions. Samples are limited in the amount of photons they can tolerate before photobleaching and photodamage (Laissue *et al*. 2017). Overall, for super-resolution microscopy techniques, trade-offs must be made between different performance metrics within their performance envelopes to meet the requirements of applications. Deep learning techniques provide a potentially transformative solution to enhance performance of super-resolution microscopy techniques and to push their performance envelopes (Belthangady and Royer 2019). Because image reconstruction is at the core of all super-resolution microscopy techniques, we focus on examining recent advances in using deep learning to advance computational reconstruction of super-resolution microscopy images.

### Deep learning-based enhancement of spatial resolution

Interestingly, the term “super-resolution” was first coined for natural images and is defined as overcoming resolution limits of optical imaging systems by image processing (Farsiu *et al*. 2004; van Ouwerkerk 2006; Yang and Huang 2017). This definition applies to biological super-resolution microscopy as well. However, what qualifies as “super-resolution” for microscopy images is strictly defined by the diffraction limit of ~200 nm. In contrast, what qualifies as “super-resolution” for natural images is more loosely defined and is often judged by human perception. Research on super-resolution for natural images has a long history that dates back at least to 1980s (Yang and Huang 2017). Conventional super-resolution techniques developed before the rise of deep learning techniques are examined in several reviews (Farsiu *et al*. 2004; van Ouwerkerk 2006; Yang and Huang 2017). However, it is deep learning that has enabled transformative performance advances (Anwar *et al*. 2020; Wang *et al*. 2020c). A wide variety of deep learning-based super-resolution techniques have been developed for natural images (Anwar *et al*. 2020; Wang *et al*. 2020c; Yang *et al*. 2019). Among them, single-image super-resolution techniques that require just one input image have been extensively studied (Dong *et al*. 2016; Lim *et al*. 2017; Yang *et al*. 2019) and have recently been used for enhancing the resolution of fluorescence microscopy images (Fang *et al*. 2021; Wang *et al*. 2019a).

The implementation of deep learning-based super-resolution techniques for both natural images and fluorescence microscopy images follow the same supervised learning paradigm that consists of two steps: training data preparation and model training. In training data preparation, paired and aligned low-resolution and high-resolution images of the same FOV are produced by physical experiments, computer simulation, or a mixture of both. In model training, the paired images are used to train DNNs to learn the mapping between the low-resolution image domain as the input domain and the high-resolution image domain as the output domain. After the models are trained, they are used to transform an input of low-resolution images into an output of high-resolution images. In this way, deep learning enables computational reconstruction of synthetic high-resolution images. This process is presumably more convenient and cost-effective than physical acquisition of real high-resolution images. Specifically, for fluorescence microscopy images, deep learning-based super-resolution techniques provide a computational solution that is potentially transformative in enhancing spatial resolutions and overcoming the diffraction limit if properly implemented.

To date, a significant number of studies have reported using deep learning to enhance spatial resolutions in image reconstruction for biological super-resolution microscopy. Overall there are two application scenarios. Under the first scenario, deep learning is used directly to reconstruct super-resolution microscopy images of higher resolutions from raw images. For example, Nehme and colleagues have reported using DNNs to enhance the performance of single-molecule localization for the reconstruction of STORM images in 2D and 3D (Nehme *et al*. 2018, 2020). Deep learning achieves higher localization accuracy than conventional point spread function (PSF) fitting under high fluorophore density and low SNR with real-time speed and no parameter tuning for 2D STORM (Nehme *et al*. 2018). They extend their work to 3D STORM by combining PSF engineering with deep learning-based single-molecule localization and PSF pattern recognition (Nehme *et al*. 2020). Indeed, deep learning is well suited for the recognition of complex patterns of engineered PSFs and has achieved superior detection accuracy and speed for image reconstruction in several other studies, *e*.*g*., Zelger *et al*. (2018) and Zhang *et al*. (2018). Under the second scenario, deep learning is used directly to transform low-resolution microscopy images to high-resolution microscopy images, similar as in super-resolution for natural images. For example, Wang and colleagues trained a GAN model to perform the transformation across different modalities, such as from low-NA widefield to high-NA widefield, from confocal to STED, and from conventional TIRF to TIRF-SIM (Wang *et al*. 2019a). Fang and colleagues used a U-Net type model to enhance the resolutions of electron microscopy images and fluorescence microscopy images (Fang *et al*. 2021). Qiao and colleagues used two deep learning models for enhancing the resolutions of SIM under low signal-to-noiseratios (SNRs) and long intervals of imaging, respectively (Qiao *et al*. 2021). Table 2 summarizes and compares key components of the reviewed studies under the two scenarios.

**Table 2:** Representative studies using deep learning to enhance spatial and temporal resolution of super-resolution microscopy

Application	Model	Imaging modality	Network architecture	Loss function	Training data	Performance metric	Reference
Enhancement of spatial resolution	DeepSTORM	2D-STORM	Deep-STORM	MSE + L1 regularization	Synthetic and experimental	MSE	Nehme *et al*. 2018
		3D-STORM with engineered PSF	DeepSTORM3D	MSE + Weighted overlap measure	Synthetic	Jaccard Index, RMSE	Nehme *et al*. 2020
	smNET	3D-SMLM with engineered PSF	Customized ResNet	MSE	Synthetic and experimental	MSE	Zhang *et al*. 2018
	−	3D-STORM with engineered PSF	VGG16	L1 loss	Synthetic	L1 error	Zelger *et al*. 2018
	−	Widefield, point-scan confocal, TIRF, STED	GAN (U-Net)	MSE + SSIM	Paired, experimental	FWHM	Wang *et al*. 2019a
	−	SEM, point-scanning confocal	Res-UNet	MSE	Paired, semisynthetic	PSNR, SSIM, FRC, NanoJ-Squirrel	Fang *et al*. 2021
	DFCAN, DFGAN	SIM	DFCAN DFGAN	MSE + SSIM (DFCAN) MSE + SSIM + BCE (DFGAN)	Paired, experimental	NRMSE, MS-SSIM, decorrelation	Qiao *et al*. 2021
Enhancement of temporal resolution	ANNA-PALM	PALM	pix2pix (GAN)	MSE + MS-SSIM + Weighted L1 loss	Paired, experimental and synthetic	MS-SSIM	Ouyang *et al*. 2018
	−	Spectroscopic SMLM	Customized ResNet	MSE	Experimental	FWHM, MS-SSIM	Gaire *et al*. 2020
BCE: binary cross-entropy; FRC: Fourier ring correlation; FWHM: full width half maximum; MSE: mean square error; PSNR: peak signal-to-noise ratio; RMSE: root mean square error; SEM: scanning electron microscopy; SSIM: structural similarity; MS-SSIM: multiscale structural similarity							

Overall, the studies examined above have successfully demonstrated the use of deep learning to enhance microscopy image resolutions across multiple modalities, including widefield microscopy, confocal microscopy, SMLM, SIM, and transmission electron microscopy, confirming the generalization capability and versatility of the approach. However, these studies also have some important limitations. First, the generation of artifacts has been reported in all the studies. Currently, there is no systematic solution to this problem. Second, performance metrics used for the reconstruction of super-resolution microscopy images in these studies are often the same as those used for natural images, such as PSNR (peak signal-to-noise ratio) and SSIM (structural similarity index). These metrics may not be well suited for fluorescence microscopy images because of their differences from natural images. Third, the generalization capability and robustness of the proposed deep learning models have not been thoroughly characterized in the studies. These limitations will be further discussed later.

### Deep learning-based enhancement of temporal resolution

Because of the basic principle of their image formation, SMLM techniques require a long acquisition time, which severely limits their applications in live-cell imaging. Increasing fluorescence labeling density can accelerate SMLM but will also increase the overlap of single fluorophore signals within the diffraction limit. Nehme and colleagues have shown that deep learning is capable of localizing single fluorophores at higher densities (Nehme *et al*. 2018, 2020). Still, the allowed density has an upper limit. In addition to increasing labeling density, a variety of chemical and physical strategies have been proposed to overcome the limitation of SMLM techniques in temporal resolution. Brighter fluorophores, stronger laser and faster cameras have all been tried (Huang *et al*. 2013; Jones *et al*. 2011; Lin *et al*. 2015). However, the fast acquisition may lower image quality while strong laser may induce photobleaching and photodamage. Overall, none of these strategies address the fundamental constraint of SMLM, namely large numbers of single molecules must be localized for faithful image reconstruction. To this end, several studies have tried to reduce the number of localized single molecules that are required using compressed sensing (Chen *et al*. 2020; Gu *et al*. 2014) and sparse support (Ovesný *et al*. 2014). Recently, DNNs have shown excellent performance and great potential in reconstructing SMLM images from sparse data (Gaire *et al*. 2020; Ouyang *et al*. 2018). Ouyang and colleagues used a customized pix2pix GAN to reconstruct high-resolution SMLM images from sparse raw images of localized single-molecules (Ouyang *et al*. 2018). In comparison, Gaire and colleagues used a simpler residual learning architecture for reconstructing high-resolution SMLM images from sparse data of up to three color channels (Gaire *et al*. 2020).

**Figure 3:**
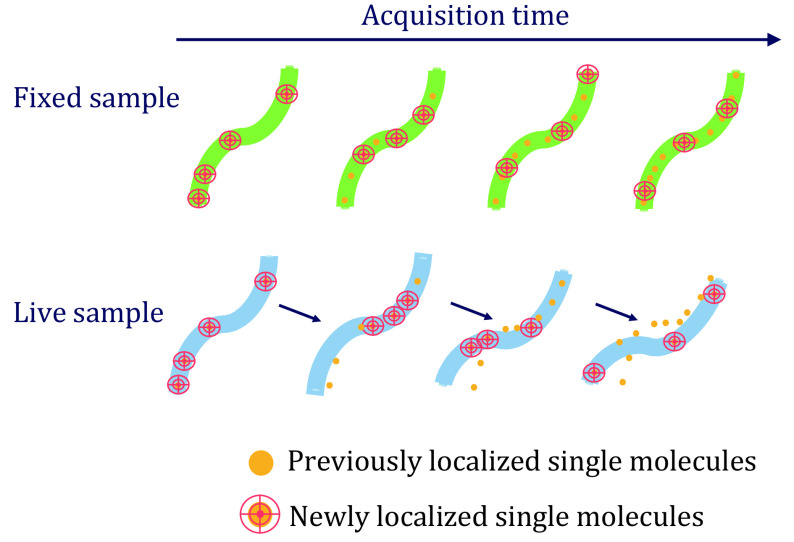
A cartoon illustration of single-molecule localization on a curvilinear structure in a fixed cell versus a live cell. In the fixed cell, localized single-molecule positions are samples of the same static underlying structure. In the live cell, localized single-molecule positions are samples of the varying underlying structure at different time points

So far, we have focused on examining deep learning techniques for the reconstruction of single super-resolution images from sparse data. In live-cell imaging, acquired videos have substantial spatial and temporal continuity. Deep learning has also been demonstrated to utilize the continuity information in videos to enhance spatial and temporal resolution of confocal and light-field microscopy (Fang *et al*. 2021; Wagner *et al*. 2021). However, specifically for SMLM, deep learning faces new challenges in assigning localized single fluorophores to moving structures. As illustrated by the cartoon example in Fig. 3, direct application of the reconstruction strategy developed for fixed samples to live samples will result in reconstruction error, and the error in single image reconstruction will propagate over time in image sequences. Simply reducing exposure time will result in a lower density of localized single fluorophores. Solving this problem requires new methods that effectively utilize the spatial and temporal continuity of acquired images.

### Deep learning-based noise reduction

Signal-to-noise ratio (SNR) is an important quality metric for fluorescence microscopy images. Low SNR images often cause difficulties in image reconstruction. Noise in fluorescence microscopy images typically is introduced by three sources: the microscope optical system, the image sensor, and background autofluorescence. Increasing laser power is a direct way to improve the SNR but will also increase the likelihood of photobleaching and photodamage. Using algorithms to reduce noise in fluorescence images provides an important noninvasive strategy. Classical denoising algorithms have been used to enhance SNR of fluorescence images under low laser power (Boulanger *et al*. 2010; Buades *et al*. 2005; Luisier *et al*. 2011). Recent studies show that deep learning-based methods have superior performance over classical methods (Weigert *et al*. 2018). So far, deep learning-based denoising has been used in image acquisition of several super-resolution microscopy modalities, including SIM (Jin *et al*. 2020), STED (Li *et al*. 2020), and SMLM (Möckl *et al*. 2020). Typically, DNNs are trained with pairs of low SNR and high SNR images that are collected from real experiments or simulations. Then they are used to denoise fluorescence images acquired under low illumination intensity. These denoising methods are particularly useful for studying fast dynamic events or fragile specimens. It is expected that deep learning based denoising will be widely adopted in super-resolution microscopy to increase SNR and to reduce photodamage of biological samples.

### Other applications of deep learning

Multicolor super-resolution microscopy uses different fluorescence probes to reveal multiple molecular structures at the same time. However, fast multicolor imaging leads to overlap of emission spectra, which in turn causes channel mixing and reduces the final resolution. Deep learning-based methods are used in separating mixed spectra during image acquisition in recent studies (Gaire *et al*. 2020; Hershko *et al*. 2019; Jones *et al*. 2011). These methods have shown excellent performance in reducing color cross-contamination, accelerating image acquisition, and increasing the final resolution of reconstructed images. In addition, deep learning has found many other successful applications in fluorescence microscopy. For example, a common strategy for 3D fluorescence microscopy is to use specialized optics and engineered PSF. A recent study has shown that deep CNN can extract depth information from a single 2D image to reconstruct a 3D image of the sample (Wu *et al*. 2019). This may bring in a new approach for 3D super-resolution microscopy. As another example, conventional SIM imaging applies structured illumination on the specimen and uses customized algorithms to reconstruct final super-resolution images from multiple acquired raw images. Deep learning methods have been shown to reduce the number of raw images required by up to five-fold and to be able to reconstruct images under extremely low light conditions (Jin *et al*. 2020). Compared to conventional SIM methods, deep learning-based SIM reconstruction methods have substantial advantages in reducing photo-damage, reducing required raw data, and reducing acquisition time. These methods have the potential to become widely adopted.

## DISCUSSION AND OUTLOOK

Today, models with tens of millions of parameters are common in deep learning (Cheng *et al*. 2017). Extremely large models have also started to be utilized. For example, the GPT-3 model, developed initially for natural language processing with 175 billion parameters (Brown *et al*. 2020), has also been used for computer vision tasks such as image synthesis. The enormous numbers of parameters, which far exceed those of traditional image processes and computer vision algorithms, are one of the key factors that give deep learning models the power to handle challenging image processing and computer vision tasks (Leshno *et al*. 1993; Lu *et al*. 2017). In practice, in addition to the numbers of model parameters, several other factors should be considered when choosing deep learning models for biological super-resolution microscopy. Model accuracy is often the most important factor. Improvement in model accuracy is usually the main goal pursued by related deep learning studies. Deeper neural networks generally tend to provide better accuracy. At the same time, however, they require more computing resources, and their processing speed is slower. For simple tasks, a lightweight model with satisfactory performance may suffice. In addition to model accuracy and computing cost, generalization capability and robustness of models are also key factors to be considered. They ensure that models will work reliably under different or changing conditions in practice. The availability of training data is another important factor to be considered. Overall, comprehensive and balanced consideration of these factors is required in selecting deep learning models for biological super-resolution microscopy.

In addition to model selection, it is important to consider the differences between fluorescence images and natural images in developing deep learning techniques for biological super-resolution microscopy. Some of the differences have been discussed above. As another example of the differences, information within the individual color channels, *e*.*g*., RGB of natural images, is often correlated. In contrast, crosstalk between different color (*i*.*e*. wavelength) channels is usually avoided in the acquisition of fluorescence microscopy images. This difference is one of the key factors that make fluorescence images easier to analyze than natural images. The development of super-resolution techniques for natural images has a long history that lasts more than four decades. It is undoubtedly necessary and beneficial to learn from this history in developing super-resolution techniques for fluorescence microscopy, as demonstrated in studies such as Wagner *et al*. (2021), Wang *et al*. (2019a) and Weigert *et al*. (2018). In the meantime, however, it is important to consider the distinct properties of fluorescence microscopy and the physical principles of fluorescence microscopes. For example, a recent study has shown that deep learning in the Fourier domain outperforms previous state-of-the-art techniques in enhancing spatial resolution for SIM (Qiao *et al*. 2021).

Overall, as demonstrated by the studies reviewed above, deep learning has great potential in pushing the performance envelope of super-resolution microscopy. In the meantime, however, it also faces critical technical challenges. Currently, to minimize or eliminate artifacts in the reconstruction of super-resolution images is a key challenge (Hoffman *et al*. 2021). These artifacts will generate misleading results and negatively impact related research (Hoffman *et al*. 2021). Overcoming this challenge will be crucial to the future development of deep learning techniques for super-resolution microscopy. Although progress has been made (Culley *et al*. 2018), the characterization of artifacts remains an open problem. Generation of artifacts is actually a common issue in solving inverse problems such as image reconstruction (McCann *et al*. 2017). Looking into the future, we expect that this issue will be gradually resolved through the synergy of multiple measures, including but not limited to rigorous experimental control, incorporation of realistic physical models, and improvement in the design of DNN architectures and loss functions. To verify findings of super-resolution microscopy using different experimental techniques is also crucial in addressing the issue of artifacts.

Another technical challenge is to ensure the generalization capability and robustness of DNNs for super-resolution microscopy. DNNs trained on images collected on selected microscopes under selected conditions should perform well on images collected on other microscopes under other conditions (Caicedo *et al*. 2019). Fluctuations in imaging conditions are common in fluorescence microscopy, especially in live cell imaging. Deep-learning models are data driven and may be sensitive to such fluctuations. In fact, if neural networks are not trained properly, their performance can collapse under such fluctuations (Chai *et al*. 2018). To ensure the interpretability of DNNs for super-resolution microscopy is also a key challenge in real-world applications (Zhang and Zhu 2018). Currently, deep learning lacks a rigorous theoretical foundation (He and Tao 2020) for the in-depth understanding of its basic properties such as generalization, robustness, and interpretability. In fact, the challenges of ensuring generalization, robustness, and interpretability of DNNs are common for all deep learning applications, not just super-resolution microscopy. We expect that they will be gradually overcome by advances in the general theory and practice of deep learning as well as customized solutions for super-resolution microscopy. Overall, despite the challenges, we expect that deep learning will play an important and potentially transformative role in advancing super-resolution microscopy as a new generation of light microscopy technology.

## Conflict of interest

Tianjie Yang, Yaoru Luo, Wei Ji and Ge Yang declare that they have no conflict of interest.
